# Dynamic External Fixation for Interphalangeal Comminuted Fractures With Mallet Injury

**Published:** 2021-03-08

**Authors:** Margaret Luthringer, Naji Madi, Amanda Chow, Ashley Ignatiuk

**Affiliations:** ^a^Division of Plastic Surgery; ^b^Department of Orthopaedics, Rutgers New Jersey Medical School, Newark

**Keywords:** dynamic distraction and external fixation, pilon, interphalangeal fracture-dislocation, ligamentotaxis, mallet

## DESCRIPTION

A 26 year-old man presented with gunshot wounds to the left ring and small fingers. The physical examination revealed open, comminuted fractures of the middle phalanges of the left ring and small fingers; an extensor lag at the distal interphalangeal joint (DIP) of the small finger suggested a terminal tendon injury. X-ray imaging demonstrated a pilon fracture of the ring finger middle phalanx base with mid-shaft comminution. The small finger middle phalanx head sustained a comminuted fracture with bicondylar involvement. After debridement in the operating room, we fabricated dynamic external fixation devices for stabilization of the fractures of the ring and small fingers; dermatotenodesis was performed to address the mallet injury. The devices were removed at 6 weeks, where minimal stiffness was noted at the ring DIP and proximal interphalangeal joint (PIP) of the small finger during active ranging. The small finger DIP demonstrated active flexion to 30° at that time with no extensor lag.

## QUESTIONS

1. What are the indications and contraindications for dynamic external fixation for finger fracture-dislocations?
2. What are the benefits and disadvantages associated with dynamic external fixation for finger fractures?
3. How were the dynamic external fixation devices constructed in this case?
4. In what way was the external fixation device fabricated for the small finger in this case novel?

## DISCUSSION

Dynamic distraction and external fixation (DDEF) is indicated for unstable PIP fracture-dislocations and pilon fractures of the middle phalanx.^1^^,^^2^ This treatment is particularly useful when closed reduction yields a congruent joint that proves dynamically unstable (typically seen when >40% of the articular surface is involved).^1^ Open reduction internal fixation techniques may be employed adjunctively for large fracture fragments.^1^^,^^3^^,^^4^ Volar plate or hemi-hamate arthroplasty may be performed concurrently to enhance dynamic stability of dorsal dislocations associated with volar lip fracture and comminution.^1^^,^^5^^,^^6^ Pilon-type fractures, defined by total articular surface involvement, and even severely comminuted fractures of the mid-shaft are well suited for DDEF, as it facilitates reduction of fracture fragments via ligamentotaxis from the surrounding soft tissues.^1^^,^^6^^,^^7^ Authors have also described the utility of DDEF for severely comminuted fractures of the DIP and metacarpophalangeal joints.^3^ DDEF is contraindicated for injuries associated with severe soft-tissue loss (compromising ligamentotaxis), chronic injury or severe arthritis, and segmental fractures involving the proximal or middle phalangeal head.^2^

DDEF maintains fracture alignment while allowing early active and passive ranging of the involved joints.^1^^,^^6^^,^^7^ This is paramount in severe phalangeal injuries prone to contracture with immobilization.^1^ Early motioning mitigates joint stiffness and promotes cartilage healing. The benefits of immediate motion for joint pliability compensate for intra-articular step-offs common in healed pilon injuries.^1^ Furthermore, the DDEF construct imposes minimal trauma on the soft tissues.^6^^,^^7^ Other benefits of DDEF include low cost, ease and quickness of device fabrication, and favorable functional outcomes.^2^ Ruland et al^5^ reported a mean final arc of motion of 88° for his series of 34 patients with PIP DDEF. All patients returned to baseline levels of activity.^5^ Disadvantages of the treatment include a learning curve with construction and placement as well as bulkiness.^2^ Pin-site infections have been reported to occur in up to 25% of patients; this can necessitate early removal of the fixator, jeopardizing fracture healing and alignment.^1^

The authors prefer to use the push-traction DDEF design described by Gaul and Rosenberg,^7^ with tension provided by Kirschner wires only.^2^ After reduction has been achieved, one double-ended 0.045 in K-wire is placed at the center of rotation of the P1 head. It is critical to confirm accurate placement with lateral fluoroscopy before advancing the wire to prevent bone injury from multiple attempts. The wire should appear as a single dark dot in the center of the phalangeal head on lateral view. Advance the wire through the opposite cortex and soft tissue until the wire flanking the finger is equal lengths. Repeat this at the P2 head so that the wires are parallel. Use a heavy needle driver to bend the distal wire into a dorsal hook on each side; the first bend should be 0.5 to 1 cm distal to the DIP. The second bend of the hook should be 1 cm proximal to the first. Trim wires. Bend the proximal wire 90° on each side and flip distally to engage the proximal wires into the dorsal hooks.^1^^,^^2^^,^^7^

The authors improvised a DDEF construct to simultaneously address the patient's small finger severely comminuted P2 head and shaft fracture (with total intra-articular surface involvement) and terminal tendon injury. Given the severity of comminution, the P2 fracture would benefit from longitudinal traction. The mallet finger required immobilization in extension.^8^ We first placed one 0.045 in K-wire transversely through the P2 head to stabilize the bicondylar fracture after open reduction. We then fashioned a DDEF construct as previously described with the proximal wire through the P1 head and the distal wire through the P3 base. The middle wire was affixed to the proximal wire dorsally. This technique promoted immediate motion of the PIP joint, while immobilizing the DIP after dermatotenodesis.

## SUMMARY

The authors constructed a push-traction DDEF device to provide reduction stability while allowing early motion of the ring finger PIP for our patient's P2 pilon fracture. We created a novel DDEF device allowing PIP motion while immobilizing the DIP for the patient's concomitant P2 head/shaft comminuted fractures and mallet injury.

## Figures and Tables

**Figure 1:**
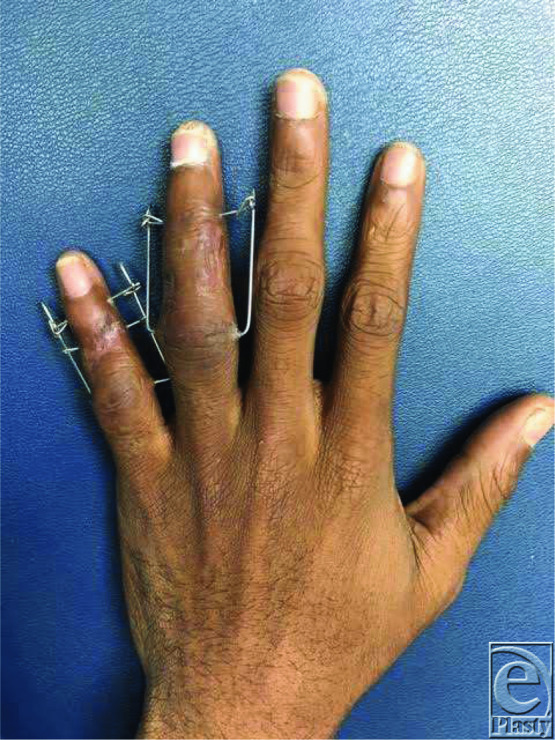
Patient 6 weeks after dynamic distraction and external fixation device placement.

**Figure 2:**
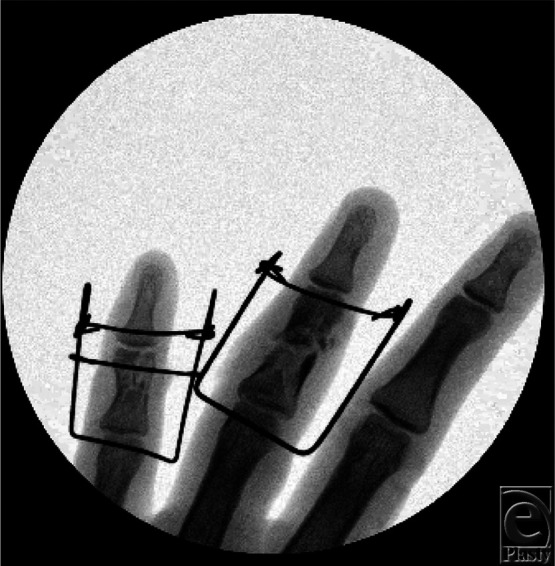
Intraoperative fluoroscopy of dynamic distraction and external fixation devices.

**Figure 3:**
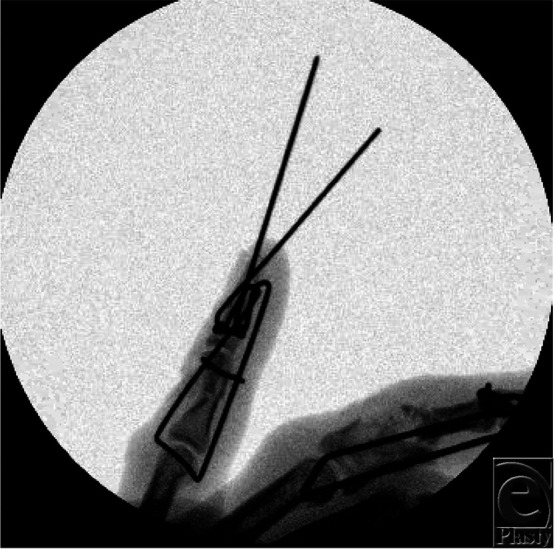
Intraoperative oblique fluoroscopy view of the small finger novel dynamic distraction and external fixation device.
